# Iron Supplementation and Copper Homeostasis: Evidence for Potential Copper Depletion, Biomarker Challenges, and Clinical Implications

**DOI:** 10.3390/nu18193117

**Published:** 2026-09-22

**Authors:** Jisu Lee, Jung-Heun Ha

**Affiliations:** Department of Food Science and Nutrition, Dankook University, Cheonan 31116, Republic of Korea

**Keywords:** iron supplementation, copper homeostasis, iron–copper interaction, ceruloplasmin, ferroxidase, micronutrient interaction, copper deficiency

## Abstract

Iron deficiency remains a major nutritional problem, particularly among women of reproductive age, pregnant women, infants, and children. Oral iron supplementation is the principal strategy for prevention and treatment, but the biological interdependence of iron and copper metabolism raises concern that prolonged or excessive iron exposure may impair copper availability in susceptible individuals. This review summarizes human and animal evidence linking iron supplementation or high dietary iron intake to altered copper homeostasis, with emphasis on underlying mechanisms, biomarker limitations, and clinical implications. Human studies report inconsistent changes in serum copper and ceruloplasmin, suggesting that baseline mineral status, physiological demand, iron dose, treatment duration, inflammation, and biomarker selection may influence responses. Animal studies provide stronger mechanistic evidence that high iron intake can reduce intestinal copper absorption and copper-dependent ferroxidase activity, promote tissue iron sequestration, and contribute to anemia, impaired growth, cardiac enlargement, dyslipidemia, gastrointestinal alterations, and disrupted maternal–offspring copper transfer. However, the clinical relevance of these findings remains uncertain because standardized diagnostic criteria and sensitive biomarkers for marginal copper deficiency are lacking. Iron intervention should remain guided by confirmed iron status, while dose, duration, and treatment response warrant careful consideration. Evaluation of copper status may be particularly relevant when hematological recovery is inadequate despite sufficient iron stores or when additional risk factors for copper deficiency are present. Prospective human studies incorporating functional biomarkers are needed to identify susceptible populations and clinically relevant exposure conditions.

## 1. Introduction

Iron deficiency represents one of the most widespread nutritional disorders globally, affecting a significant portion of the world’s population. An estimated 1.2 billion individuals are affected, with women of reproductive age, pregnant women, and young children being particularly susceptible [1]. Factors such as menstrual blood loss, increased physiological demands during pregnancy, and rapid growth contribute to the vulnerability of these groups [2,3]. Despite the greater dietary availability in developed countries, iron deficiency remains prevalent, while limited access to nutrient-rich foods exacerbates the condition in developing regions [1,4]. Clinical manifestations extend beyond anemia, encompassing fatigue, impaired immunity, and diminished work capacity [3,5,6,7]. Furthermore, iron deficiency during early life is linked to long-term cognitive and developmental impairments [8,9,10]. These widespread consequences underscore the necessity of maintaining adequate iron status as a global public health priority.

Iron depletion impacts multiple organ systems and life stages. In children, inadequate iron status is frequently associated with impaired cognitive function and delayed psychomotor development [8,10,11]. In adults, diminished oxygen transport capacity leads to reduced physical performance and increased fatigue [3,5,7,12]. During pregnancy, iron deficiency elevates the risk of maternal morbidity and adverse birth outcomes. At the population level, decreased productivity and increased healthcare expenditure impose substantial socioeconomic burdens [13,14]. Consequently, public health strategies have prioritized the prevention and treatment of iron deficiency, with nutritional interventions serving as crucial components of disease prevention programs [1,12,15,16]. The combined health, developmental, and economic consequences justify the continued implementation of effective strategies to prevent iron deficiency across vulnerable populations [1,6,12].

Oral iron supplementation and food fortification are pivotal strategies for addressing iron deficiency and preventing related complications in patients with IDA [1,16,17,18]. Although the advantages of these interventions are well established, evidence indicates that high-dose or prolonged iron exposure may diminish copper availability in specific conditions [19,20,21,22]. Copper insufficiency is significant because copper-dependent proteins are integral to intestinal iron export, systemic iron mobilization, mitochondrial function, antioxidant defense, and erythropoietic iron utilization [12,23,24,25]. Consequently, the disruption of copper homeostasis may affect several processes essential for normal iron metabolism, even when iron intake is adequate [23,24]. Individuals with sufficient copper reserves may tolerate supplemental iron without noticeable disruption, whereas those with marginal copper status, low dietary copper intake, impaired absorption, rapid growth, pregnancy-related demand, or prolonged high-dose iron exposure may be more susceptible to iron-induced copper deficiency.

Human studies investigating the interaction between iron supplementation and copper status have yielded inconsistent results [21,26,27]. Some studies have observed decreased circulating copper concentrations during iron or combined iron and folic acid supplementation, particularly during pregnancy, whereas others have reported no adverse changes or increases in serum copper and ceruloplasmin levels after iron repletion [21,26,27]. Inconsistent circulating measurements do not preclude biologically relevant interactions, as conventional copper indices may not reliably detect early, marginal, or tissue-specific disturbances in copper homeostasis in the liver [28]. Serum copper and ceruloplasmin levels are also affected by inflammation, estrogen status, pregnancy, infection, and liver function [27,29,30]. Therefore, this review evaluates the evidence that iron supplementation may disrupt copper homeostasis, focusing on marginal copper deficiency, limitations of current biomarkers, and distinguishing between experimentally demonstrated effects and mechanistic inferences.

## 2. Literature Search Strategy and Study Selection

We carried out a literature search in PubMed to find studies that involve both humans and animals and that deal with how oral or dietary iron exposure affects copper homeostasis. The final search finished on 16 September 2026 and included all records in PubMed from its beginning up to that date. We used search strategies for human studies and animal studies to account for differences in study design, exposure conditions and outcomes. Our human search used the terms: (“iron supplementation” OR “oral iron” OR “iron supplements” OR “iron-folate” OR “iron folic acid”) AND (“copper” OR “ceruloplasmin” OR “copper absorption” OR “copper status” OR “copper-zinc superoxide dismutase” OR “CuZn-SOD”) AND Humans [MeSH Terms]. We counted a study as eligible for our human evidence if it looked at oral iron supplementation or exposure and measured at least one copper-related outcome. Those outcomes could be serum or plasma copper, ceruloplasmin level or activity copper-dependent enzyme activity, copper absorption or copper in breast milk. Studies that did not look at iron or did not measure any copper-related outcome were left out. We also excluded studies that used iron, reviews, editorials, comments, case reports or studies that did not provide original data. If a study examined iron dose, response or iron absorption but did not directly measure any copper-related outcome we kept it only as background information when it helped explain how iron exposure interacts with intestinal minerals.

Our animal search used these terms: (“ iron” OR “high-iron” OR “dietary iron overload” OR “iron overload” OR “excess dietary iron” OR “carbonyl iron”) AND (“copper” OR “copper deficiency” OR “ceruloplasmin” OR “ferroxidase” OR “copper absorption”) AND Animals [MeSH Terms]. Our main animal review focused on studies that exposed animals to high iron through diet or oral routes and that directly measured copper status, copper absorption, ceruloplasmin or ferroxidase activity, copper-dependent enzymes, tissue copper levels or the interaction between iron and copper. We left out studies that used parenteral iron, used animals other than rodents or did not have elevated dietary or oral iron exposure. We also found rodent studies that did not measure copper directly but gave useful background on growth problems blood changes, liver iron build-up gut changes or metabolic issues linked to high iron. We kept these studies as background information when they were relevant.

In both searches we first screened titles and abstracts then carefully examined the reports that seemed eligible using the available abstracts and full texts. Our human search found 104 records. We examined 25 of them in detail for eligibility. After that review we excluded four reports. This left 21 reports that represent 20 human studies for our final synthesis. Our animal search found 308 records. We reviewed 23 of them closely for eligibility. After that review we excluded four reports. This gave us 19 reports for our animal synthesis.

We also looked at reference lists, from the articles and relevant reviews to spot studies that the electronic search missed. Because our structured search only used PubMed, studies that appear in other databases might have been overlooked. Therefore, we put together the evidence in a way instead of doing a quantitative analysis. The way we found and chose studies is shown in a PRISMA-style flow diagram (Figure 1).

## 3. Iron Supplementation: Clinical Indications and Exposure Contexts

Oral iron supplementation is the most common and cost-effective strategy for addressing iron deficiency [6,17,18]. Iron tablets and fortified foods are extensively utilized in both clinical and public health settings [1,12,16]. The broad implementation of supplementation has enhanced iron status in numerous populations [16,31,32]. However, these interventions often concentrate solely on iron repletion, overlooking interactions with other essential trace minerals [1,32]. Nutrient–nutrient interactions can influence absorption, distribution, and overall metabolic balance [24,33,34]. Consequently, high-dose supplementation may result in unintended physiological effects [18,19,20,35]. The homeostasis of trace minerals necessitates coordinated regulation rather than isolated nutrient replacement [33,36,37]. To optimize nutritional interventions, consideration of mineral interactions is crucial [1,32,37].

Iron supplementation varies significantly based on the dose, formulation, treatment duration, baseline iron status, and physiological condition [18,32]. Preventive supplementation, therapeutic supplementation for iron-deficiency anemia, and experimental high-iron feeding do not represent identical exposure conditions. Short-term iron repletion in deficient individuals may provide clear hematological benefits, whereas prolonged or excessive intake may increase the risk of secondary trace mineral imbalance [5,18,19,20,31,32]. This risk may be heightened when copper intake is marginal, intestinal absorption is impaired, inflammation is present, or supplementation continues without biochemical monitoring. Therefore, interpreting high-dose iron exposure requires careful differentiation between clinically indicated correction of deficiency and sustained intake beyond physiological requirements [18,19,20,32]. A comprehensive review of iron supplementation should consider both the necessity of restoring iron status and the potential for excessive luminal or systemic iron to interfere with copper-dependent metabolic pathways [23,24].

## 4. Biological Basis of Iron–Copper Interaction

Iron homeostasis cannot be fully understood without considering the trace minerals that facilitate iron transport and utilization [12,23,24]. Iron uptake, storage, export, oxidation, and delivery to erythroid tissues necessitate coordinated interactions among intestinal transporters, hepatic regulatory signals, plasma carrier proteins, and metal-dependent enzymes [38,39,40,41]. Copper is integral to this network, as copper-dependent ferroxidases convert exported ferrous iron into a form that can bind transferrin [23,42,43]. Consequently, impaired copper availability may lead to a functional blockade of iron mobilization, even when total body iron stores are sufficient [23,24,42]. Physiologically, both iron excess and copper deficiency may result in the same clinical outcome: reduced iron availability for erythropoiesis [24,42,44]. Therefore, integrating the biology of iron and copper is crucial for understanding paradoxical anemia under conditions of high iron exposure.

### 4.1. Intestinal Absorption and Transporter-Level Interactions

The efficiency of iron absorption is significantly affected by the chemical form of dietary iron [41,45]. Heme iron, predominantly sourced from animal products, is absorbed via specialized pathways with relatively high efficiency [41,45]. Conversely, non-heme iron, commonly found in plant-based foods and supplements, is transported across the apical membrane of enterocytes primarily by divalent metal transporter 1 (DMT1) [40,41,43,45]. Once internalized, intracellular iron is either stored in ferritin or exported into the circulation through ferroportin, the sole mammalian iron exporter [39,40,41]. The exported ferrous iron is subsequently oxidized to ferric iron to facilitate its binding to transferrin [23,42,43]. The absorption of non-heme iron is also modulated by luminal conditions and systemic regulatory signals [41,45,46]. Phytates and polyphenols inhibit absorption, whereas ascorbic acid enhances bioavailability [47,48,49]. Consequently, intestinal regulation allows iron absorption to adapt to physiological demand.

Intestinal copper absorption is primarily mediated by copper transporter 1 (CTR1) located on the apical membrane of enterocytes [24,36,50]. Following uptake, intracellular copper is bound by specific chaperone proteins that mitigate oxidative damage and direct copper to appropriate cellular compartments [24,36,50]. Copper may be stored in metallothionein or transported to the trans-Golgi network by ATPase copper transporting alpha (ATP7A), which facilitates copper incorporation into cuproenzymes and export into the systemic circulation [24,36,50].

Given that the intestine is a major site for potential interactions between iron and copper, understanding the transport mechanisms of both metals is crucial for interpreting nutrient interactions [19,20,24]. Although DMT1 plays a central role in non-heme iron uptake, its direct contribution to copper transport is less clearly established compared to the roles of CTR1 and ATP7A [24,36,50,51]. Therefore, transporter-level competition should be considered a potential mechanism rather than a universally confirmed pathway [20,24,52]. High luminal iron may indirectly influence copper homeostasis by altering enterocyte metal handling, affecting shared regulatory processes, or reducing copper absorption efficiency, particularly during excessive iron exposure or marginal copper availability.

### 4.2. Copper-Dependent Ferroxidases: Ceruloplasmin and Hephaestin

Copper plays a critical role in iron metabolism by acting as a cofactor for ferroxidase, hephaestin, and ceruloplasmin. These enzymes are responsible for the oxidation of ferrous iron (Fe^2+^) to ferric iron (Fe^3+^), a process essential for loading iron onto transferrin and its subsequent systemic transport [23,42,43]. Although both proteins facilitate iron mobilization, they differ in their physiological locations and functions [23,42]. Hephaestin is primarily expressed in intestinal enterocytes, where it supports dietary iron absorption by promoting iron export across the basolateral membrane following ferroportin-mediated efflux [23,42,43]. Conversely, ceruloplasmin, a ferroxidase present in circulation and associated with tissues, is involved in the mobilization of iron from hepatocytes, macrophages, and other storage sites [23,39]. Thus, hephaestin is crucial for intestinal iron absorption, whereas ceruloplasmin is more integral to systemic iron distribution and recycling [23,42,43]. Copper deficiency can impair ferroxidase activity, hindering the conversion of Fe^2+^ to Fe^3+^ and reducing the efficiency of transferrin loading [23,24,42]. This impairment leads to decreased iron export and mobilization, resulting in tissue iron sequestration and reduced iron availability for erythropoiesis [23,42,53,54]. This mechanism elucidates how copper deficiency can mimic iron deficiency by causing functional iron restriction, even when total body iron stores are sufficient or elevated. Importantly, ceruloplasmin concentration and its ferroxidase activity are not always synonymous, as protein abundance may not accurately reflect enzymatic function when copper incorporation or inflammatory status is altered [29,30].

### 4.3. Hepcidin–Ferroportin and Epo–Erfe Axes

Systemic iron homeostasis is meticulously regulated by the hepcidin–ferroportin axis [39,55,56,57]. Hepcidin, a peptide hormone predominantly synthesized in the liver, regulates iron export by binding to ferroportin, the principal cellular iron exporter found in enterocytes, macrophages, and hepatocytes [39,55,56]. The binding of hepcidin induces internalization and degradation of ferroportin, thereby diminishing iron release into the circulation [39,55,58]. Elevated hepcidin levels restrict intestinal iron absorption and macrophage iron recycling, whereas decreased hepcidin levels facilitate iron mobilization [39,55,56]. The hepcidin–ferroportin axis is responsive to iron stores, erythropoietic demand, inflammation, and hypoxic stress, thereby coordinating iron absorption, storage, and recycling [39,55,56].

During anemia or erythropoietic stress, iron mobilization is further modulated through the erythropoietin–erythroferrone–hepcidin axis [59,60,61]. Reduced oxygen delivery enhances renal erythropoietin (Epo) production, which stimulates the expansion of erythroid precursors and the production of erythroferrone (Erfe) in erythroid tissues [59,62]. Erfe subsequently suppresses hepatic hepcidin expression, permitting ferroportin-mediated iron release from enterocytes and macrophages to support hemoglobin synthesis [39,59,60]. Thus, the Epo–Erfe response functions as a compensatory mechanism that augments circulating iron availability when erythropoietic demand is heightened [59,61,62].

In conditions of high iron accompanied by impaired copper-dependent iron mobilization, the compensatory response may become decoupled from effective iron utilization. Anemia may stimulate the production of Epo and Erfe, yet tissue iron sequestration, sustained hepcidin activity, or diminished ferroxidase function may limit the release of stored iron and its loading onto transferrin [39,42,59,63]. Such conditions may result in a discrepancy between iron abundance and availability: iron remains sequestered in storage tissues, but its delivery to erythroid cells is restricted [42,63,64]. Therefore, the interpretation of hepcidin, Epo, and Erfe responses in high-iron or copper-limited states should consider copper-dependent ferroxidase capacity in addition to total iron stores.

## 5. Evidence from Human Studies

### 5.1. Effects of Iron Supplementation on Circulating Copper Indices

Research on the interaction between copper and oral iron intake is both limited and diverse, with outcomes influenced by factors such as study population, life stage, baseline mineral levels, type and dosage of iron, duration of intake, diet, and specific copper-related indicators measured (Table 1) [21,26,27,28,65,66,67,68,69,70,71,72,73] In pregnant women with high-normal hemoglobin levels, a daily intake of 50 mg of iron as ferrous sulfate was linked to reduced blood copper levels compared to a placebo [21]. Similarly, Bangladeshi infants receiving weekly iron supplementation exhibited decreased blood copper levels [66]. Conversely, other studies reported that a five-week iron supplementation increased blood copper levels, and among women with low iron status, ceruloplasmin levels also rose [26]. Investigations involving breastfed infants and adults with ileostomies indicated no adverse effects on copper absorption when iron was administered [27,73]. These findings suggest that the response of copper to oral iron intake varies across different populations.

Pregnancy represents a critical period because of substantial physiological changes that can influence blood copper levels. In a study by Ziaei et al., 66 pregnant women with normal hemoglobin levels (≥13.2 g/dL) at 13–18 weeks gestation were administered either 50 mg of iron daily as ferrous sulfate from 20 weeks until delivery or a placebo [21]. Iron intake was found to affect blood copper levels during the second and third trimesters. Another study demonstrated that women taking iron and folic acid had lower blood copper levels compared to those not taking iron [74]. Pregnancy-related factors, such as estrogen levels, inflammation, physiological adaptation, infections, liver function, and complications like preeclampsia, can each influence blood copper and iron levels [28,65,70,71,72]. Therefore, a reduction in copper levels due to iron intake does not necessarily indicate copper deficiency.

Studies involving non-pregnant women also yield varied results. In non-anemic women with low iron levels, a five-week iron supplementation increased blood copper levels, and among those with iron, ceruloplasmin levels also increased [26]. These findings suggest that correcting iron levels may enhance or maintain copper levels rather than invariably diminishing them. The initial levels of iron and copper may account for the variability in copper level changes observed among individuals taking iron supplements.

Research involving infants indicates that outcomes vary based on specific indicators assessed. In a study involving infants from Bangladesh, one-week iron supplementation resulted in reduced blood copper levels [66]. Conversely, another study involving breastfed infants found that iron supplementation did not significantly affect copper absorption efficiency [27]. Furthermore, in breastfed infants, administering iron at a dosage of 1 mg per kg of body weight daily resulted in decreased levels of copper–zinc superoxide dismutase in the blood compared to a placebo, despite an increase in blood copper levels as the infants aged [75]. These findings that intestinal iron intake does not invariably hinder copper absorption and may be influenced by factors such as the quantity of iron consumed, duration of intake, individual’s physiological state, initial mineral levels, and specific mineral involved.

Additional insights were provided by evidence of short-term iron exposure. In adults with ileostomies, the administration of 100 or 400 mg iron did not significantly impair copper absorption, although zinc absorption was affected [73]. This finding indicates that intestinal iron intake does not invariably hinder copper absorption and may be influenced by factors such as the quantity of iron consumed, duration of intake, individual’s physiological state, initial mineral levels, and specific mineral involved.

Overall, human studies have not consistently demonstrated that oral iron supplementation leads to copper deficiency. The available evidence is heterogeneous in study design, population, iron regimen, and copper-related outcome measures, which limits direct comparison across studies and precludes a meaningful quantitative synthesis. Instead, the response of copper to iron intake appears to be contingent upon factors such as the population group, initial mineral levels, life stage, type and quantity of iron, duration of iron intake, and the indicators used to evaluate copper levels. Some studies have reported alterations in copper levels or copper-dependent enzyme activity, while others have observed no change or even increased levels when addressing iron deficiency. Consequently, a reduction in copper levels or copper-dependent enzyme activity should not be automatically interpreted as an indication of copper deficiency. Moreover, the absence of changes in copper levels does not necessarily imply an absence of issues related to copper availability in the body.

### 5.2. Influence of Iron Dose and Dosing Schedule

Most oral iron trials have predominantly focused on optimizing iron absorption, with limited evaluation of copper [68,69,73,76,77,78,79,80]. Research conducted by Moretti, Stoffel, and colleagues demonstrated that larger or more frequent oral iron doses elevate hepcidin concentrations, diminish fractional absorption from subsequent doses, and result in a greater proportion of administered iron remaining in the intestinal lumen [68,69,73,76,77]. In iron-depleted young women, oral iron doses of ≥60 mg elevated serum hepcidin levels for up to 24 h and reduced fractional absorption from subsequent doses by approximately 35–45% [68,76]. Increasing the administered dose from 40 to 240 mg only increased the absolute amount of absorbed iron from 6.7 to 18.1 mg, indicating that absorption did not increase proportionally with the dose [68].

Related studies have indicated that alternate-day dosing results in greater fractional iron absorption than consecutive day dosing, whereas twice-daily divided dosing increases hepcidin levels without offering a clear absorption advantage over once-daily morning dosing [80]. In women with iron-deficiency anemia, 100- and 200-mg oral iron doses also elevated serum hepcidin for approximately 24 h, and fractional absorption was lower with consecutive-day dosing than with alternate-day dosing [68,73,79]. Although these findings do not demonstrate copper depletion, they delineate an exposure pattern that may increase the potential for intestinal mineral interaction. Prolonged high-dose regimens may increase luminal iron exposure without proportionally enhancing iron repletion because the administered dose does not directly correspond to the absorbed dose [68,69,73,76,77].

Clinical dose-comparison studies also advise against assuming that higher oral iron doses necessarily confer greater therapeutic benefits than lower doses [67]. In a randomized study of patients older than 80 years with iron deficiency anemia, daily elemental iron doses of 15, 50, or 150 mg for 60 days produced similar increases in hemoglobin and ferritin levels [67]. However, gastrointestinal adverse effects occurred more frequently at higher doses than at lower ones [67]. The findings suggest that increasing the oral iron dose may offer limited additional benefits in certain clinical settings, while increasing gastrointestinal intolerance and the amount of unabsorbed iron remaining in the intestinal lumen.

Collectively, the available human data suggest that repeated higher-dose exposure may enhance hepcidin-mediated suppression of absorption, gastrointestinal adverse effects and luminal iron accumulation. Although the evidence does not directly establish iron-induced copper deficiency, it raises concerns that unnecessary, prolonged, or high-dose oral iron exposure may create intestinal and systemic conditions relevant to trace-mineral interactions. Therefore, future trials should prospectively assess copper-related outcomes across different iron formulations, doses, and dosing schedules rather than inferring trace mineral safety solely from hematologic responses. Importantly, the high dietary iron exposures commonly used in animal models substantially exceed typical clinical supplementation and differ in formulation, duration, and nutritional context. Accordingly, adverse copper-related effects observed in these models should be interpreted primarily as mechanistic evidence rather than directly extrapolated to standard therapeutic iron doses in humans.

### 5.3. Persistent Anemia and Copper-Dependent Iron Mobilization

The lack of a standardized diagnostic framework for copper deficiency is a significant consideration when interpreting human literature. Persistent anemia during iron therapy is often attributed to factors such as poor adherence, ongoing blood loss, inflammation, malabsorption, or an incorrect diagnosis, which should be evaluated initially [6,17,29,81]. However, the possibility of iron-associated copper limitation should be considered when anemia persists despite adequate or elevated iron stores, particularly during prolonged higher-dose therapy or in individuals with low copper intake or impaired gastrointestinal absorption [18,23,24,42,72,82,83,84]. Additional iron may be ineffective or counterproductive if the limiting defect involves copper-dependent iron mobilization rather than iron supply [18,23,24,42,83,84].

Differentiating iron-deficiency anemia from copper-related impairment of iron utilization is crucial because of the distinct underlying nutritional defects [29,81,82,84]. Iron-deficiency anemia results from insufficient iron availability due to depleted stores, inadequate intake, or increased loss [6,17,29,81]. Conversely, copper deficiency or reduced copper-dependent ferroxidase activity may impair iron mobilization despite the presence of stored iron [18,23,24,42,82,84]. Potential biochemical indicators include alterations in serum copper, ceruloplasmin concentration, ceruloplasmin ferroxidase activity, and indices of iron sequestration [24,28,70,71,82,85]. Circulating copper-related markers should be interpreted with caution, as factors such as inflammation, pregnancy, estrogen status, infection, and liver function can influence their concentrations [28,70,71,85]. Copper-related mechanisms should not be presumed in all cases of iron-refractory anemia but may be considered when conventional explanations are insufficient [23,42,82,83].

### 5.4. Susceptible Populations and Marginal Copper Status

Iron–copper interactions may be particularly relevant in individuals with marginal copper status prior to supplementation. Marginal copper deficiency may not manifest the hematologic or neurologic symptoms of overt deficiency and may remain undetected by serum copper or ceruloplasmin concentration [28,70,71,85]. Conditions such as pregnancy, infancy, rapid growth, restrictive diets, bariatric or gastrointestinal disease, prolonged enteral nutrition, inflammation, and concurrent high zinc exposure may further deplete copper reserves or complicate biomarker interpretation [23,28,42,70,71,72,84,85,86]. Current human evidence does not establish that routine iron therapy commonly induces overt copper-deficiency anemia. However, available findings support the hypothesis that supplemental iron may unmask or exacerbate marginal copper deficiency in susceptible individuals. Testing this hypothesis requires assessment of baseline copper status, dietary copper intake, functional copper biomarkers, and treatment duration.

### 5.5. Interpretation and Limitations of Human Evidence

Unchanged serum copper should not be considered definitive evidence that copper homeostasis is unaffected. A study may fail to detect copper depletion due to insufficient exposure duration, adequate baseline copper stores in participants, or the insensitivity of the selected marker to marginal deficiency. Conversely, a decline in serum copper should not automatically be interpreted as clinically significant copper deficiency. Current human evidence suggests that iron supplementation can alter copper-related physiology under certain conditions, but the prevalence, threshold dose, susceptible phenotype, and clinical significance remain unresolved [21,26,27,65,66,67,72].

**Table 1:** Human evidence evaluating copper-related outcomes during oral iron supplementation or oral iron exposure.

Population/Study	Study Design	Iron (Fe) Regimen	Copper (Cu)-Related Outcomes	Key Findings	Main Methodological Limitation
Pregnant women, *n* = 66; [21]	Randomized, double-blind, placebo-controlled trial	50 mg/day elemental Fe as ferrous sulfate from gestational week 20 to delivery	Serum Cu, Zn	Serum Cu was lower in the Fe group during the second and third trimesters	Selected high-normal-Hb population; serum Cu does not establish tissue Cu deficiency
Pregnant women, *n* = 57; [74]	Observational supplemented vs. unsupplemented comparison	Daily iron–folate from gestational weeks 11–15 to 33–35	Serum Cu	Supplemented women had lower median serum Cu than unsupplemented women	Nonrandomized; unable to distinguish a decrease with supplementation from physiological increase in controls
Pregnant women; [87]	Longitudinal supplemented vs. unsupplemented study	100 mg Fe/day during the final 3 months of pregnancy	Serum Cu; breast-milk Cu	Fe supplementation had no significant effect on maternal serum or milk Cu	Physiological pregnancy/lactation changes strongly influence circulating and milk Cu
Pregnant women; [88]	Observational iron-supplement users vs. nonusers	Antenatal Fe supplementation; regimen varied	Serum Cu	Serum Cu was lower overall in supplement users, although Cu increased during pregnancy in both groups	Nonrandomized; supplementation indication, diet, and baseline status may confound findings
Pregnant women; [89]	Controlled supplementation study	200 mg ferrous sulfate/day ± vitamin A for 15 wk	Serum Cu	Serum Cu was not significantly affected by supplementation	Relatively small groups; one arm included vitamin A
Pregnant women; [26]	Randomized controlled micronutrient trial	Folic acid + Fe, with other micronutrient arms	Serum Cu	Serum Cu increased during pregnancy; no clear adverse Cu effect attributable to the Fe-containing regimens	Multi-micronutrient design limits attribution specifically to iron
Lactating women, *n* = 145; [90]	Observational comparison according to supplementation history	Fe during pregnancy and/or breastfeeding	Serum Cu, ceruloplasmin, breast-milk Cu	Maternal Cu-related indices differed according to supplementation exposure	Observational; diet, baseline status, and supplementation history may confound interpretation
Non-anemic iron-depleted women, *n* = 15; iron-adequate comparison, *n* = 12; [91]	Before–after supplementation study	Oral Fe for 5 wk	Serum Cu; ceruloplasmin; erythrocyte SOD	Serum Cu increased after Fe repletion; ceruloplasmin also increased in participants with the lowest ferritin	Small sample; no untreated Fe-depleted control
Iron-depleted/iron-deficient women; [76]	Prospective supplementation and discontinuation study	320 mg elemental Fe/day for 12 wk, followed by 12 wk without supplementation	Serum Cu	Fe therapy increased ferritin but did not decrease serum Cu	High stated dose; only 25 participants completed full follow-up
Healthy 1-y-old infants, *n* = 291; [92]	Randomized placebo-controlled trial	30 mg Fe as ferrous sulfate for 3 mo	Serum Cu	No significant difference in serum Cu between Fe and placebo groups	Serum Cu only; well-nourished infant population
Healthy term breastfed infants, *n* = 77; [93]	Double-masked randomized trial	7.5 mg/day elemental Fe from 1–6 mo	Plasma Cu; erythrocyte SOD	No significant treatment-related biochemical difference in Cu-related indices	Cu was not the primary endpoint
Healthy term breastfed infants, *n* = 214; [75]	Randomized trial	1 mg/kg/day from 4–9 mo or 6–9 mo vs. placebo	Plasma Cu; erythrocyte CuZn-SOD	CuZn-SOD activity was lower after supplementation from 4–9 mo, whereas plasma Cu increased with age	Functional marker change does not establish clinical Cu deficiency
Same parent infant trial, *n* = 25 substudy; [77]	Stable-isotope absorption substudy	1 mg/kg/day Fe according to parent trial	Stable-isotope Cu absorption	Prior Fe supplementation did not significantly alter Cu absorption	Small substudy; not an independent study population
Low-birth-weight infants, *n* = 55; [94]	Randomized trial	13.8 mg, 7 mg, or no elemental Fe/day	Plasma Cu; erythrocyte SOD	Plasma Cu did not differ, but erythrocyte SOD activity was lower in the high-Fe group at 20 wk	Functional enzyme endpoint; clinical relevance uncertain
Low-birth-weight infants, *n* = 58; [95]	Randomized comparison of two Fe levels	Formula containing 13.4 vs. 20.7 mg Fe/L	Plasma Cu; SOD	Plasma Cu tended to be lower with high-iron formula at 12 mo; SOD and other antioxidant indices were also assessed	Both groups received Fe; no placebo/no-iron control
Healthy term infants, *n* = 53; [96]	Randomized Fe supplementation vs. no supplementation	10 mg elemental Fe/day for 2 mo	Intraerythrocytic Cu; SOD	No significant differences in intraerythrocytic Cu or SOD between groups	Short intervention; healthy Fe-replete infants
Bangladeshi infants, *n* = 333; [66]	Double-blind randomized community trial	20 mg Fe weekly, Zn weekly, combined Fe + Zn, or control for 6 mo	Serum Cu	Weekly Fe supplementation decreased serum Cu; combined supplementation did not materially change the individual effects	Cu was a secondary endpoint
Children with IDA, *n* = 25; [97]	Comparative oral iron treatment study	Ferrous sulfate vs. ferric hydroxide-polymaltose for 6 mo	Plasma Cu; ceruloplasmin	Ceruloplasmin decreased during ferrous Fe therapy; plasma Cu was transiently lower than with ferric iron	Small sample; comparison between Fe formulations rather than placebo
Children of short stature, *n* = 30; [98]	Randomized iron + zinc, zinc alone, or placebo	12 mg Fe/day + 12.5 mg Zn/day for 1 y	Serum Cu; hair Cu	Cu was measured as part of the trace-element panel; study primarily evaluated growth and combined Fe + Zn supplementation	Fe was co-administered with zinc, limiting attribution specifically to iron
Children receiving oral iron; [99]	Oral iron treatment study	Ferrous compound; detailed regimen not available in PubMed abstract	Serum Cu; ceruloplasmin	Study directly assessed changes in serum Cu and ceruloplasmin during oral iron therapy	Limited methodological information available from the PubMed abstract

Abbreviations: Cu, copper; CuZn-SOD, copper–zinc superoxide dismutase; Fe, iron; Hb, hemoglobin; IDA, iron-deficiency anemia; mo, month(s); SOD, superoxide dismutase; wk, week(s); y, year(s); Zn, zinc; vs., versus. Iron doses and formulations are presented as reported in the cited studies; direct comparisons should consider whether the stated amount refers to elemental iron or the iron compound. Copper-related outcomes included circulating Cu concentrations, ceruloplasmin, Cu-dependent enzyme activity, and stable-isotope Cu absorption, depending on the study. Changes in serum or plasma Cu do not necessarily indicate tissue Cu deficiency or clinically significant copper depletion, and functional markers such as ceruloplasmin or SOD should be interpreted in the context of study design, physiological status, and baseline nutritional status. Evidence from observational or multi-micronutrient studies should also be interpreted cautiously because effects cannot always be attributed specifically to iron. Numbers in brackets correspond to references in the source list.

## 6. Evidence from Animal Models

### 6.1. Growth Impairment and Copper-Related Anemia

High dietary iron intake, particularly at levels far exceeding nutritional requirements, has been associated with impaired growth in developing rodents. Severe high-iron exposure, including diets containing approximately 8800 ppm iron, has produced marked reductions in body weight gain and, under some experimental conditions, increased mortality in weanling Sprague-Dawley rats and C57BL/6 mice [100,101,102,103,104,105]. Although iron is essential for physiological development, excessive exposure can induce marked growth impairment despite adequate caloric intake [101,102,106]. Several high-iron models also developed anemia despite substantial hepatic iron accumulation and elevated systemic iron availability [100,101,102,103,104]. These abnormalities were frequently accompanied by reduced copper status and impaired copper-dependent iron mobilization, supporting a role for copper-related functional iron restriction [107,108]. Because copper is required for normal iron export and utilization, reduced copper availability may promote iron sequestration in storage tissues while limiting its delivery for hemoglobin synthesis. However, copper-deficiency anemia is unlikely to be the sole explanation for impaired growth, as growth abnormalities have also been reported in some high-iron models without direct evidence of copper depletion. Collectively, the findings suggest that high iron exposure may impair growth through a combination of direct iron toxicity and disturbances in copper-dependent iron metabolism.

### 6.2. Dietary Iron Overload Models with Limited Copper Assessment

Models of dietary iron overload, such as carbonyl iron, high-iron purified diets, and oral ferrous sulfate exposure, are extensively employed to induce experimental iron loading in rodents. As outlined in Table 2, prior research frequently documented reduced body weight or impaired weight gain, particularly in growing rodents, alongside significant hepatic iron accumulation and, in certain instances, elevated circulating iron levels. Hematological responses varied among studies, with hemoglobin and hematocrit often remaining unchanged or unmeasured [100,101,102]. Consequently, high dietary iron exposure may elicit systemic effects beyond hepatic iron deposition, although the mechanisms underlying growth impairment, altered organ weights, bone abnormalities, gastrointestinal damage, or metabolic dysfunction were not clearly elucidated in many early models.

A significant limitation of the early studies was the infrequent evaluation of copper-related endpoints. Many experiments did not report dietary copper content, tissue or serum copper concentrations, ceruloplasmin concentration or activity, or ferroxidase activity [61,105,109,110,111,112,113,114]. Consequently, growth impairment and anemia-related outcomes in carbonyl iron and high-iron diet models cannot be retrospectively attributed to copper deficiency [61,105,109,110,111,112,113,114]. Nonetheless, similarities between the reported phenotypes and established responses to copper deficiency support the hypothesis that altered copper absorption, distribution, or cuproenzyme function may have contributed to some of the observed effects [100,101,102,115].

Studies that directly manipulated dietary iron and copper provide stronger evidence for the proposed interaction. In weanling rodents, high-iron feeding impaired growth and was associated with anemia-related abnormalities, reduced hepatic copper, decreased serum ceruloplasmin activity, and cardiac hypertrophy [100,101,102]. Increasing dietary copper prevented or mitigated several abnormalities, suggesting that high-iron exposure may produce a mixed phenotype involving iron loading and copper-dependent functional impairment [100,101]. Subsequent mechanistic studies demonstrated that high iron reduced intestinal copper absorption and disrupted systemic copper homeostasis [100,102]. Copper supplementation restored copper status and corrected several high-iron-associated abnormalities without necessarily reducing tissue iron accumulation.

Later investigations of oral and dietary iron exposure further demonstrated that growth and metabolic outcomes should be interpreted in the context of trace-mineral interactions [100,106,116,117,118,119]. High-dose ferrous sulfate exposure in pre-weanling rats resulted in failure to thrive, hepatic iron accumulation, and altered hepatic copper and zinc concentrations [116]. Other high-iron diet models reported reduced weight gain, smaller body size, lower fat mass, altered ferritin or hepcidin expression, impaired glucose responses, metabolic changes in adipose tissue and liver, mild duodenal inflammation, or alterations in the gut microbiota [106,117,118,119]. Although copper-dependent mechanisms were not consistently assessed, these findings indicate that high iron exposure can affect growth and metabolism through pathways extending beyond the amount of iron deposited in the liver [106,116,117,118,119]. Future studies on dietary iron overload should therefore include copper-related endpoints to distinguish direct iron effects from secondary disturbances in copper metabolism.

**Table 2:** Animal studies of high dietary or oral iron exposure and associated copper-related and physiological outcomes.

Iron (Fe) Exposure	Rodent Model	Exposure Period	BW/Growth	Hb/Hematology	Fe/Mineral Status	Copper (Cu)-Related Assessment	Main Interpretation
2% carbonyl Fe [113]	Weanling Wistar rat	8–10 wk	NC	NM	Hepatic Fe ↑; serum Fe ↑	NM	High Fe loading altered intestinal growth/morphology and transferrin receptor-related measures.
0.84% dietary Fe [120]	Adult C57BL/6 mouse	16 wk	↓	NC	NM	NM	Fe-enriched diet model associated with metabolic dysfunction and adipose effects.
3% dietary Fe [118]	6-wk C57BL/6 mouse	16 wk	↓	NM	Hepatic Fe ↑; serum hepcidin ↑; adipose Fe ↑	NM	Fe supplementation was linked to altered Fe, glucose, and adipose metabolism.
1–2% carbonyl Fe [109]	Adolescent F344 rat	5 wk	↓	NM	Hepatic Fe ↑	NM	Dietary Fe overload model used in a hepatic injury/carcinogenesis context.
1.25–2.5% carbonyl Fe [112]	Weanling Wistar rat	23 wk	↓	NM	Hepatic Fe ↑	NM	Long-term carbonyl Fe feeding used to model hepatic Fe overload in hepatocarcinogenesis studies.
2–3% dietary Fe [111]	Weanling Sprague-Dawley rat	3 wk	↓	↑	Hepatic Fe ↑; hepatic Cu NC with 3% Fe and ↓ with 2% Fe	Hepatic Cu	Dietary Fe overload caused marked hepatic Fe accumulation and altered hepatic metal homeostasis; hepatic Cu was reduced in the 2% Fe-overload model but not in the 3% Fe-overload model.
3% dietary Fe [110]	Weanling Porton rat	10 wk	↓	NM	Hepatic Fe ↑	NM	Dietary Fe overload model in growing rats, with reduced BW and hepatic Fe ↑.
2% dietary Fe [121]	Weanling Sprague-Dawley rat	3 wk	↓	NC	Hepatic Fe ↑	NM	Weanling rat Fe-overload model, with reduced BW and hepatic Fe increased.
5000 or 8000 mg Fe/kg diet [114]	Weanling CD-1 mouse	7 wk	↓	Hct NC	Hepatic weight ↑; Fe deposition index ↑; hepatic Fe ↑	NM	Prolonged excess dietary Fe caused hepatic Fe accumulation and biochemical hepatotoxicity.
1500–10,000 μg carbonyl Fe/g diet [105]	B6C3F1 mice, C5YSF1 mice, F344 rats	12 wk	Strain-dependent; NC or ↓	NM	Hepatic nonheme Fe ↑	NM	Genetic background modified growth toxicity and hepatic response to Fe overload.
HFe approximately 8800 ppm with low, adequate, or high Cu [101]	Weanling Sprague-Dawley rat	5 wk	↓	Hb ↓ with low/adequate Cu; prevented with high Cu	Serum/tissue Cu ↓; ceruloplasmin activity ↓; serum nonheme Fe ↑ and transferrin saturation ↑ with high Cu	Serum/tissue Cu; ceruloplasmin activity; Cu rescue	HFe caused systemic Cu deficiency; extra Cu prevented growth impairment, anemia, cardiac hypertrophy, and low ceruloplasmin activity.
HFe approximately 8800 ppm vs. adequate Fe approximately 80 ppm, with variable Cu [102]	Weanling mouse	5 wk	↓	↓	Hepatic Cu ↓; serum ceruloplasmin activity ↓	Hepatic Cu; ceruloplasmin activity; intestinal Cu handling	Excess dietary Fe impaired Cu homeostasis and was investigated as an intestinal Cu-absorption defect.
HFe > 100-fold excess or adequate Fe; Cu in drinking water (20 mg/L Cu) [100]	Weanling C57BL/6 mouse	4–7 wk	↓; partially normalized with Cu	Hb restored; anemia reversed	Nonheme Fe ↑ in serum and tissues with HFe; tissue Fe NC with Cu; intestinal ^64^Cu absorption ↓ ~60% with HFe	^64^Cu absorption; tissue Cu; Cu rescue	Cu supplementation restored Cu status and corrected many HFe-related pathologies.
Ferrous sulfate at 10, 30, or 90 mg Fe/kg BW/day [116]	Pre-weanling Lewis rat pups	PD2–9	NC with FS-10/30; ↓ with FS-90	WBC ↑ with FS-90; Hb not central	Hepatic Fe ↑; hepatic Cu ↓; hepatic Zn ↑ with FS-90	Hepatic Cu and Zn	Growth impairment at the highest dose was associated with hepatic Fe loading, inflammatory signaling, and trace-mineral disturbances.
High-Fe diet, 1250 mg Fe/kg food [119]	C57BL/6 mice	12 wk	BW gain ↓; body size ↓	NM	Serum ferritin **↑**; Fe metabolism altered	NM	Adipogenesis ↓; lipolysis ↑ in liver/adipose tissue; slight duodenal inflammation and gut microbiota changes.
HFe, FeSO_4_ 9.9 g/kg diet [117]	C57BL/6J mice exposed from gestation to adulthood	To 30 wk postnatal	BW ↓; fat mass ↓	NM	Hepatic Fe **↑**; hepcidin **↑**	NM	Altered ferritin expression, adipocyte differentiation, and triglyceride metabolism may impair normal growth.
1.25 g Fe/kg diet [106]	C57BL/6J mouse	9 wk	↓	NM	Hepatic Fe **↑**	NM	Hepatic gluconeogenesis ↓; glucose responses impaired.
AIN-93G diets containing 1×, 4×, 9.5×, 18.5×, 38×, or 110× Fe [122]	Weanling Sprague–Dawley rats	7 wk for 110× Fe; 8 wk for all other groups	Final BW ↓ ~40% with 110× Fe	Hb and Hct ↓ with 110× Fe	Tissue Fe ↑ dose-dependently; hepatic and splenic Cu ↓ at higher Fe intakes	Serum ceruloplasmin activity; hepatic, splenic, renal, and bone Cu	The severity of impaired growth, anemia, and Cu-homeostasis disturbances increased with dietary Fe concentration.
35 vs. 217 mg Fe/kg diet with marginal Cu (2.0 or 2.5 mg Cu/kg) [123]	Weanling Sprague-Dawley rat	42 d (Exp. 1); 96 d (Exp. 2)	NM	No adverse hematologic changes	Hepatic Fe ↑; cardiac/hepatic/plasma Cu ↓ under marginal-Cu conditions	Plasma, hepatic and cardiac Cu; ceruloplasmin activity	High dietary Fe reduced several Cu-status indices when dietary Cu was marginal, supporting the possibility that Fe excess increases Cu requirements.
Normal vs. 10-fold higher Fe intake (approximately 35 vs. 350 mg added Fe/kg diet), with normal or high Cu/Zn [124]	Weanling Wistar rat	4 wk	NM	NM	Plasma Cu ↓ with high Fe in rats receiving normal Cu; tissue Cu largely NC	Plasma, liver, spleen, kidney and tibia Cu	High Fe lowered plasma Cu under normal-Cu conditions, although tissue Cu concentrations were largely unchanged; high Zn and Fe effects were additive.
20 g/kg carbonyl Fe ± Cu in drinking water (350 mg/L) [125]	Developing rat; dams and offspring	Gestational 20 d to postnatal 63 d	NM	NM	Hepatic Fe ↑ 17–30-fold; hepatic, kidney and plasma Cu generally ↓ with Fe loading	Tissue/plasma Cu; organ Fe uptake and deposition	Dietary Fe excess altered Cu distribution, while concurrent Cu loading modified Fe uptake and tissue deposition in an age- and organ-dependent manner.
High-Fe diet vs. control diet [126]	Nagase analbuminaemic and Sprague-Dawley rats	NM	NM	Baseline strain-related Hb/Hct differences	Kidney Cu ↓; Cu absorption and biliary Cu excretion ↓ with high Fe	Tissue/plasma Cu; ceruloplasmin; Cu absorption; biliary Cu excretion	High dietary Fe reduced renal Cu and impaired Cu absorption and excretion, although the specialized analbuminaemic model limits generalization.
180 vs. 45 mg Fe/kg diet with Cr(III) supplementation [127]	Wistar rat	NM	NM	NM	Hepatic Cu ↓ 16%; kidney Cu ↓ 32%; spleen Cu ↑ 76% with Fe excess	Tissue Cu and Zn	Fe oversupply altered tissue Cu distribution independently of Cr(III), providing direct evidence that moderate Fe excess can disturb Cu status.
54 vs. 226 μg Fe/g diet with deficient or adequate Cu and sucrose or cornstarch [128]	Sprague-Dawley rat	19 d	NM	Hct ↓ most markedly in Cu-deficient/high-Fe/sucrose rats	Cu deficiency → tissue Cu ↓ and hepatic Fe ↑; tissue Cu not significantly altered by Fe level	Tissue Cu; ceruloplasmin; apparent Cu absorption	High Fe aggravated anemia under Cu-deficient conditions, particularly with sucrose, but did not independently reduce tissue Cu concentrations.
38 vs. 191 μg Fe/g diet ±1% ascorbic acid, with deficient or adequate Cu [129]	Weanling Sprague-Dawley rat	20 d	NM	Hb ↓ 23% in Cu-deficient rats receiving high Fe plus ascorbic acid	^59^Fe utilization altered; cytochrome c oxidase activity ↓ with Cu deficiency	Functional Cu-dependent cytochrome c oxidase; ^59^Fe utilization	Elevated dietary Fe aggravated impaired Fe utilization and anemia when Cu status was deficient, indicating strong dependence of Fe effects on Cu availability.
35 vs. 1500 mg Fe/kg diet with adequate or deficient Cu [130]	Weanling Long-Evans rat	4 wk	NM	NM	Hepatic Fe ↑ with Fe overload; liver/plasma Cu and hepatic CuZn-SOD ↓ with Cu deficiency	Liver/plasma Cu; hepatic CuZn-SOD	Dietary Fe overload increased oxidative stress markers but did not consistently exacerbate the biochemical effects of Cu deficiency, providing useful null/interaction evidence.
50 vs. 300 μg Fe/g diet with 0.6 or 5.6 μg Cu/g diet [131]	Weanling and Lewis rats	42 d	NM	Sex-dependent Hb/Hct responses; high Fe did not uniformly worsen anemia	Serum/liver Cu and ceruloplasmin ↓ with Cu deficiency regardless of Fe level	Serum/liver Cu; ceruloplasmin activity	Dietary Fe modified some hematologic responses to Cu deficiency but did not substantially alter the primary Cu-deficiency phenotype.
44 vs. 87 μg Fe/g diet with adequate or deficient Cu [132]	Weanling Sprague-Dawley rat	NM	NM	NM	Intestinal Cu ↓ with Cu deficiency; intestinal Fe not significantly altered by doubling dietary Fe	Intestinal Cu and Fe; β-carotene dioxygenase activity	High Fe alone had limited effects on intestinal Cu status, whereas Cu deficiency strongly altered intestinal Fe handling and enzyme activity.
Low, normal, or high Fe (0.9, 9.0, or 90 mmol Fe/kg diet) with adequate or deficient Cu and starch/fructose [133]	Weanling Sprague-Dawley rat	4 wk	NM	NM	Intestinal Fe ↑ with dietary Fe and Cu deficiency; Cu status determined by dietary Cu	Intestinal/hepatic Fe and Cu-related interaction; β-carotene dioxygenase	Increasing dietary Fe and Cu deficiency jointly altered intestinal Fe-dependent enzyme activity, supporting an intestinal Fe–Cu interaction.
53 vs. 506 μg Fe/g diet with adequate or deficient Cu and differing dietary fat [103]	Weanling Sprague-Dawley rat	NM	NM	Severe anemia in Cu-deficient/high-Fe rats	Hepatic Fe ↑ in Cu-deficient/high-Fe group	Cu-deficient vs. adequate dietary conditions; Fe-dependent pathological phenotype	High dietary Fe markedly aggravated anemia, cardiomegaly, hepatomegaly, and hyperlipidemia under Cu-deficient conditions.
Low, adequate, or fortified Fe (3.4, 8.6, or 15.7 mmol Fe/g diet) with adequate or deficient Cu [104]	Weanling Sprague-Dawley rat	6 wk	NM	Mortality ↑ with higher Fe in Cu-deficient groups	Hepatic Fe ↑ with Fe intake	Dietary Cu-deficient vs. adequate conditions; hepatic Fe–Cu interaction	Increasing hepatic Fe burden aggravated hypercholesterolemia and hypertriacylglycerolemia in Cu-deficient rats.
High-Fe/low-Cu dietary condition with Cu supplementation [134]	Sprague-Dawley rat	NM	NM	NM	High Fe/low Cu associated with disturbed cholesterol-related pathways; Cu supplementation mitigated changes	Dietary Cu manipulation and Cu rescue	High Fe combined with low Cu altered hepatic cholesterol-related transcription, whereas Cu supplementation attenuated the metabolic disturbance.

Abbreviations: BW, body weight; Cr(III), chromium(III); Cu, copper; CuZn-SOD, copper–zinc superoxide dismutase; d, day(s); Exp., experiment; Fe, iron; FeSO_4_, ferrous sulfate; FS, ferrous sulfate; Hb, hemoglobin; Hct, hematocrit; HFe, high-iron diet; NC, no significant change; NM, not measured or not reported; PD, postnatal day; WBC, white blood cell count; wk, week(s); Zn, zinc; vs., versus; ^59^Fe, iron-59 radiotracer; ^64^Cu, copper-64 radiotracer; ↑, increased relative to the corresponding control group; ↓, decreased relative to the corresponding control group. Iron exposures are presented as reported in the cited studies and include different iron preparations, dietary concentrations, and units; therefore, direct comparisons should consider iron form and elemental iron content. Copper-related outcomes included circulating and tissue Cu concentrations, ceruloplasmin activity, Cu-dependent enzyme activity, Cu absorption, and responses to Cu supplementation, depending on the study. Changes in circulating or tissue Cu concentrations do not necessarily indicate clinically significant Cu deficiency, and Cu-related outcomes should be interpreted in the context of dietary Cu status, iron dose, experimental model, and study duration. Evidence from studies combining high Fe exposure with Cu deficiency or other mineral manipulations should also be interpreted cautiously because the observed effects cannot always be attributed specifically to iron. Numbers in brackets correspond to references in the source list.

### 6.3. Hepatic Iron Sequestration and Ceruloplasmin Dysfunction

In models characterized by high iron feeding, concurrent hepatic copper depletion, diminished ceruloplasmin-associated ferroxidase activity, hepatic iron accumulation, and reduced hemoglobin levels have been frequently documented [100,101,102]. The correlation between elevated iron intake and systemic anemia in animal models may be partially attributed to the dysfunction of copper-dependent ferroxidase ceruloplasmin (Cp) [100,101,102,107,108]. Cp is essential for the oxidation of ferrous iron (Fe^2+^) to ferric iron (Fe^3^), a critical process that enables iron binding to transferrin and its subsequent transport from storage tissues to the bone marrow for erythropoiesis [107,108]. High iron feeding decreases hepatic copper concentrations, which may impede the hepatic synthesis of functional holo-ceruloplasmin [101,102]. The resulting reduction in serum ferroxidase activity can obstruct iron export, leading to iron accumulation in hepatocytes and macrophages [107,108]. This sequestration accounts for the observation that animals on high-iron diets exhibit low hemoglobin levels despite excessive hepatic or systemic iron stores; the iron, although present, is biologically less available for red blood cell production due to compromised copper-dependent enzymatic support [100,101,102,108].

### 6.4. Cardiac Hypertrophy Associated with Copper Limitation

Cardiac hypertrophy is a significant characteristic of severe copper deficiency and has been documented in rodent models in which increased iron exposure coincides with decreased tissue copper availability [100,101,115]. Copper is crucial for cytochrome c oxidase and other pathways integral to mitochondrial energy metabolism [115,135,136]. Consequently, diminished copper availability may lead to compromised myocardial bioenergetics and compensatory cardiac remodeling [115,135,136]. In models with high iron levels, the mitigation or prevention of cardiac enlargement through copper supplementation supports the hypothesis that copper limitation contributes to the cardiac phenotype [100,101]. Nonetheless, iron-induced oxidative damage, anemia-related hemodynamic stress, and other metabolic disturbances may also influence this process [115,137,138]. Therefore, cardiac hypertrophy under high-iron conditions should be regarded as a multifactorial outcome rather than solely a result of copper deficiency.

### 6.5. Dyslipidemia and Altered Hepatic Lipid Metabolism

Animal studies have demonstrated that the metabolic effects of high iron exposure and copper deficiency extend beyond anemia [103,104,118,119,134]. Dose-response studies indicate that disturbances related to copper, such as reduced ceruloplasmin activity, may occur when dietary iron surpasses physiological needs, even before overt signs of iron toxicity are evident. However, dietary iron concentrations used in animal models should not be directly equated with human supplemental doses due to significant differences in species, absorption efficiency, dietary matrix, growth stage, and treatment duration [100,101,102]. Consequently, findings from animal studies should be regarded primarily as mechanistic evidence rather than direct dose equivalents applicable to human supplementation.

High iron intake combined with marginal copper status has been linked to dyslipidemia, particularly hypercholesterolemia. Transcriptomic analyses have associated this phenotype with coordinated changes in hepatic cholesterol metabolism, including increased expression of *Srebp2* and *Pcsk9*, which are involved in cholesterol synthesis and LDL receptor regulation, and decreased expression of *Abcg5*, which contributes to biliary cholesterol excretion [134]. The mitigation of these transcriptional alterations through copper supplementation indicates that a secondary deficiency in copper may play a role in lipid disturbances observed under high-iron conditions [134]. Thus, high iron–low copper conditions may disrupt hepatic lipid homeostasis by altering cholesterol synthesis, clearance, and excretion pathways.

### 6.6. Intestinal Copper Absorption and Gastrointestinal Alterations

The intestinal mucosa also appears to be a significant site of iron–copper antagonism. Radiotracer studies using ^64^Cu have shown that high dietary iron can attenuate the adaptive increase in intestinal copper absorption typically observed during systemic copper deficiency [100,102]. Copper absorption generally increases when body copper stores are low, but this compensatory response seems impaired in high-iron-fed animals [102]. The precise molecular mechanism remains unresolved, although excessive luminal iron may affect enterocyte copper acquisition, intracellular trafficking, or regulatory signaling [102]. Therefore, the intestine may contribute both to the development of copper limitation and to the failure of normal homeostatic mechanisms to restore copper status [102].

High-iron feeding has also been associated with structural changes in the gastrointestinal tract. Rats receiving high-iron diets exhibited shorter colons than control animals, although the biological basis of this morphological change remains uncertain [113]. Prevention of the change by copper supplementation suggests that adequate copper status may contribute to the maintenance of intestinal structure under high-iron conditions. However, the relative contributions of unabsorbed luminal iron and systemic copper limitation cannot be distinguished from the available findings. Collectively, animal evidence indicates that iron–copper imbalance may affect hepatic lipid metabolism, intestinal copper absorption, and gastrointestinal structure, supporting consideration of both hepatic and intestinal mechanisms in high-iron models.

### 6.7. Evidence from Copper Supplementation and Rescue Experiments

Research utilizing animal models indicates that the antagonistic interaction between iron and copper may affect maternal–offspring mineral transfer [139,140]. In studies involving pregnant and lactating Sprague Dawley rats, increased maternal iron exposure has been observed to reduce maternal copper availability and decrease copper concentrations in milk. Consequently, suckling pups display symptoms akin to secondary copper deficiency, such as reduced hepatic copper levels and diminished serum ceruloplasmin activity [140]. These findings imply that elevated maternal iron exposure can influence copper delivery to offspring under experimental conditions. However, as these observations are derived from animal studies, they should not be directly extrapolated to human pregnancy or lactation.

Another significant observation from animal models is that several abnormalities induced by high iron levels can be prevented or reversed through copper supplementation [100,101]. Copper supplementation, administered either through diet or drinking water, has been shown to enhance growth, reduce cardiac hypertrophy, restore hemoglobin levels, and normalize copper-dependent ferroxidase function in animals subjected to high iron intake [100,101]. These findings support the hypothesis that many adverse effects of high iron intake are mediated, at least in part, by secondary copper deficiency rather than iron excess alone [100,101,102]. Thus, experiments involving copper supplementation provide substantial mechanistic evidence for the interdependence of iron and copper metabolism, underscoring the necessity of balanced trace mineral intake to prevent unintended physiological disturbances under conditions of high iron exposure [100,101,102].

## 7. Biomarker Challenges in the Assessment of Copper Status

Iron status can be evaluated using well-established biomarkers that reflect iron stores, iron availability for erythropoiesis, and the development of anemia [6,17,67,141,142]. In contrast, copper status is more difficult to assess because no standardized staging system or single biomarker reliably identifies early or marginal copper deficiency [28,143,144,145,146]. Serum copper and ceruloplasmin are the most commonly used indices, but both are influenced by acute-phase responses, infection, inflammation, pregnancy, estrogen exposure, and liver function [86,147]. These limitations are particularly relevant in populations frequently receiving iron supplementation, including pregnant women, children, older adults, and individuals with inflammatory disorders [29,86,143,145,147]. Consequently, isolated changes in serum copper or ceruloplasmin should not be interpreted as definitive evidence of copper depletion or repletion. The principal biomarkers used to assess copper status and copper-dependent iron mobilization, together with their biological interpretation, strengths, and limitations, are summarized in Table 3.

Ceruloplasmin concentration and ceruloplasmin-associated ferroxidase activity represent related but distinct measures [86,144,147,148]. Ceruloplasmin protein concentration may increase during inflammation or estrogen exposure, whereas ferroxidase activity may provide additional information on copper incorporation and functional activity, although it is not routinely assessed in clinical practice [144,147,148]. Other proposed indicators, including erythrocyte Cu/Zn-superoxide dismutase activity, copper chaperone for superoxide dismutase, and tissue copper concentrations, may provide complementary information but have limited clinical applicability [149,150,151,152]. Hepatic copper concentration is particularly informative in experimental studies but is unsuitable for routine human assessment [143,146,151,152].

Given these limitations, copper status should be assessed using a combination of biochemical and clinical information rather than a single circulating marker. Serum or plasma copper and ceruloplasmin should be interpreted together with inflammatory status, iron indices, dietary intake, supplementation history, physiological state, and relevant clinical findings [28,86,143,144,145,146,148]. Where available, functional measures such as ceruloplasmin-associated ferroxidase activity may provide additional support when circulating copper indices are inconclusive. Longitudinal assessment may also be more informative than a single measurement, particularly when evaluating changes during prolonged iron exposure [146,148]. A practical framework for considering copper assessment during oral iron supplementation is summarized in Figure 2. Future studies should prioritize the validation of integrated biomarker panels capable of detecting marginal or functional copper deficiency across different physiological and clinical settings.

**Table 3:** Biomarkers for assessing copper status and copper-dependent iron mobilization.

Biomarker	Biological Interpretation	Main Strengths	Major Limitations and Confounders
Serum or plasma copper	Predominantly reflects copper carried by ceruloplasmin	Widely available; decreases in moderate or severe deficiency	Influenced by inflammation, infection, pregnancy, estrogen, age, sex, and liver function; limited sensitivity for marginal deficiency [28,86,143,145,146]
Ceruloplasmin protein concentration	Major circulating copper-binding protein; indirect indicator of copper status	Routinely available; useful in severe deficiency and selected copper disorders	Positive acute-phase reactant; affected by pregnancy, estrogen, inflammation, assay method, sex, and age; ceruloplasmin protein measurements may not directly reflect functional copper-loaded ceruloplasmin [28,29,143,144,145,147,153]
Ceruloplasmin oxidase or ferroxidase activity	Functional activity of copper-loaded ceruloplasmin	Mechanistically relevant to Fe^2+^ oxidation, transferrin loading, and systemic iron mobilization	Limited assay standardization and clinical availability; activity can differ from protein concentration [144,147,148]
Ceruloplasmin specific activity	Oxidase activity relative to ceruloplasmin protein mass	May help distinguish increased protein abundance from functional copper incorporation	Limited validation, reference intervals, and assay harmonization [144,147,148]
Exchangeable copper	Non-ceruloplasmin-bound exchangeable copper pool	Emerging measure of biologically available circulating copper	Better established for copper overload than marginal deficiency; method availability and reference ranges remain limited [67]
Labile serum copper	Readily exchangeable or weakly bound copper fraction	Potential sensitivity to dynamic changes in circulating copper	Early-stage evidence; uncertain nutritional interpretation and limited standardization [67]
Erythrocyte CuZn-SOD activity	Functional activity of a copper-dependent antioxidant enzyme	Responded to copper supplementation in a controlled infant study	Influenced by factors other than copper; inconsistent sensitivity; enzyme turnover may delay response [28,143,149]
Copper chaperone for SOD1	Intracellular copper-delivery protein; expression may rise during copper deficiency	Sensitive to mild copper depletion in experimental models	Evidence is predominantly from rodents and other animals; human clinical validation is insufficient [28,143,151,152]
CCS/SOD1 ratio	Combines increased CCS with reduced SOD1 during copper deficiency	Enhanced sensitivity in animal blood-cell studies	Not standardized or validated for routine human use [152]
Hepatic copper concentration	Direct tissue copper measurement	Reflects liver copper burden and is informative in mechanistic animal studies	Invasive; affected by heterogeneous hepatic distribution and liver disease; unsuitable for routine nutritional screening [143,146,151]
Hemoglobin and blood-cell indices	Detect hematologic consequences of overt copper deficiency	Routine and clinically relevant	Late and nonspecific; anemia may be microcytic, normocytic, or macrocytic; cannot identify marginal deficiency [143,145]
Ferritin and transferrin saturation	Assess stored and circulating iron availability associated with copper-dependent iron mobilization	Helps identify iron sequestration or distinguish iron depletion	Ferritin is an acute-phase reactant; values do not directly measure copper status [6,17,67,141,142]
Soluble transferrin receptor	Reflects cellular iron demand and iron-restricted erythropoiesis	Less directly affected by inflammation than ferritin	Assay and threshold variabilty; not a copper-specific marker [6,17,67,141]
C-reactive protein or other inflammatory marker	Indicates inflammatory confounding of ferritin, serum copper, and ceruloplasmin	Widely available and improves contextual interpretation	Does not correct biomarker values or directly identify copper deficiency [86,141,142,144]
Dietary and supplement history	Identifies low copper intake, high zinc exposure, high-dose iron, or prolonged supplementation	Directly addresses exposure and modifiable risks	Recall error and uncertain nutrient bioavailability [28,146]

Abbreviations: CCS, copper chaperone for SOD1; Cu, copper; CuZn-SOD, copper-zinc superoxide dismutase; Fe^2+^, ferrous iron; SOD1, superoxide dismutase 1; Zn, zinc. Exchangeable copper and labile serum copper represent operationally defined circulating copper fractions and may differ according to the analytical method used. No single biomarker reliably identifies marginal copper deficiency acroruschss all clinical settings. Copper status should therefore be interpreted using multiple biochemical and functional measures together with inflammatory markers, hematologic and iron indices, liver function, and dietary or supplement history. Reference intervals and assay results may vary according to laboratory, population, and analytical method. Numbers in brackets correspond to references in the source list.

## 8. Clinical Implications and Monitoring Strategies

Current evidence underscores the necessity of a more nuanced approach to iron supplementation, particularly in individuals undergoing prolonged or high-dose oral iron therapy [6,17,141,142]. Iron therapy remains crucial when iron deficiency is confirmed, and the findings discussed herein should not be misconstrued as evidence against appropriate iron replenishment [6,17]. Instead, they indicated that iron supplementation should be customized based on baseline iron status, treatment indication, dosage, duration, therapeutic response, and broader micronutrient context [6,17,141,142]. Currently, routine copper monitoring is not recommended for all individuals receiving iron [24,28,146,154]. However, copper-related assessments may be warranted in specific cases, such as in patients with persistent anemia despite supplementation, inadequate hematological response, adequate or elevated ferritin or transferrin saturation despite anemia, prolonged high-dose iron use, restrictive dietary patterns, gastrointestinal disorders, or other risk factors for trace mineral imbalance [24,28,71,81,146,154,155].

### 8.1. Identification of Patients Requiring Copper-Related Assessment

Routine monitoring of copper levels is not universally recommended for individuals undergoing iron supplementation [24,28,146,154]. Nonetheless, an assessment of copper status may be justified in patients exhibiting persistent anemia despite supplementation, inadequate hematological response, or anemia with normal or elevated ferritin or transferrin saturation levels [24,71,81,154]. Additional risk factors include prolonged high-dose iron intake, restrictive dietary habits, gastrointestinal disorders, impaired nutrient absorption, and other conditions associated with trace mineral imbalances [24,71,81,154,155].

Experimental evidence further supports the careful evaluation of patients requiring high-dose or long-term iron therapy [19,122]. In clinical practice, persistent anemia during iron supplementation is often attributed to inadequate iron intake or insufficient dosing [6,17]. However, copper-related impairment of erythropoiesis may mimic persistent iron deficiency, as reduced copper-dependent ferroxidase activity can hinder iron mobilization despite adequate or elevated iron stores [24,147,154]. Increasing the iron dose under such circumstances may not enhance hematological recovery and could exacerbate copper deficiency [19,122]. Therefore, persistent anemia during iron therapy should prompt an evaluation of factors such as poor adherence, ongoing blood loss, inflammation, malabsorption, incorrect diagnosis, and potential impairment of copper-dependent iron utilization [6,17,24,71,81,154].

Clinical findings suggestive of copper deficiency should be interpreted with caution [28,29,143,146,148]. Copper-related mechanisms should not be assumed in all cases of iron-refractory anemia [24,154]. However, assessment may be warranted when conventional causes have been excluded and the patient presents risk factors for marginal copper deficiency or prolonged exposure to high-dose iron [19,24,28,71,81,122,146,154].

### 8.2. Integrated Monitoring of Iron and Copper Status

An effective monitoring strategy should commence with the confirmation of iron deficiency and include periodic reassessment of the treatment response [6,17,141,142]. Essential measurements may include hemoglobin, hematocrit, serum ferritin, transferrin saturation, serum iron, total iron-binding capacity, and inflammatory markers such as C-reactive protein [6,17,141,142]. It is crucial to confirm the treatment indication, justify the prescribed dosage, regulate the treatment duration, and perform follow-up assessments to prevent prolonged high-dose exposure when clinical necessity is unsubstantiated.

In certain patients, copper-related measurements may provide supplementary information. Relevant indices may include serum copper, ceruloplasmin concentration, ceruloplasmin-associated ferroxidase activity (when available), and dietary copper intake [28,29,143,146,148,149]. The interpretation of serum copper and ceruloplasmin levels should be approached with caution, as factors such as inflammation, pregnancy, estrogen status, infection, and liver function can affect circulating concentrations [28,29,143,146,148,149]. Consequently, copper-related indices should be assessed in conjunction with iron status markers, inflammatory status, dietary information, gastrointestinal factors, iron deficiency from impaired iron mobilization or other causes of inadequate treatment response [24,28,29,143,146,148,149,154]. Longitudinal measurements relative to the baseline may provide more insight than isolated values [146,148,149].

Reevaluation of supplement formulations and clinical guidance may be warranted when iron is administered at pharmacological doses for extended periods [6,17,19,122,141,142]. Factors such as dose, formulation, dosing frequency, baseline mineral status, dietary quality, and treatment duration may influence the balance between the therapeutic benefits and unintended metabolic effects [6,17,19,122,141,142]. Future clinical recommendations should consider nutrient–nutrient interactions at both the intestinal and systemic levels and delineate the circumstances in which copper assessment or modification of the iron regimen is justified [19,24,28,29,122,143,146,147,148,149,154]. Such measures would not undermine the importance of iron repletion but could enhance the precision, safety, and long-term efficacy of iron replacement therapy [6,17,141,142].

### 8.3. Considerations for Pregnancy, Infancy, and Other Susceptible Populations

Maternal and developmental findings derived from animal models merit consideration; however, these results should not be directly extrapolated to form universal guidelines for human pregnancy and lactation [156,157]. Animal studies indicating reduced milk copper levels, hepatic copper depletion in offspring, and decreased ceruloplasmin activity in offspring suggest that maternal mineral balance may become significant under conditions of excessive iron exposure or marginal copper intake in the offspring [156,157]. Human studies are necessary to ascertain whether similar effects occur in pregnant or lactating women and to evaluate the clinical relevance of these changes in humans [21,27,87,90].

Prenatal iron supplementation should adhere to established guidelines based on iron status and the risk of the population [6,17,21]. Current evidence does not support the notion that routine prenatal iron supplementation induces a copper deficiency [21,27,87,90]. Nonetheless, pregnancy, infancy, and periods of rapid growth are characterized by increased mineral demand, and marginal copper status may increase susceptibility to iron–copper interactions. An assessment of maternal dietary intake, supplement composition, treatment dose and duration, and neonatal outcomes may help elucidate the clinical significance of this interaction.

Other potentially susceptible populations include individuals with restrictive diets, those with bariatric or gastrointestinal diseases, impaired intestinal absorption, prolonged enteral nutrition, high zinc exposure, inflammation, or marginal copper intake [24,28,71,81,146,154,155]. Evaluating copper-related parameters may be particularly informative when patients in these groups exhibit atypical hematological responses despite apparently adequate iron repletion.

### 8.4. Limitations of Routine Copper Co-Supplementation

Consequently, co-supplementation with copper should be undertaken with caution. Animal studies have demonstrated that copper supplementation can prevent or reverse various abnormalities associated with high iron exposure, such as impaired growth, reduced hemoglobin levels, cardiac enlargement, and decreased copper-dependent ferroxidase activity [19,122,156,157]. Nonetheless, these findings do not warrant universal co-supplementation with copper in humans undergoing iron therapy [24,154,158].

The optimal copper dosage, duration, target population, efficacy, and safety of combined iron–copper supplementation remain undefined. Excessive or unnecessary copper supplementation may also lead to adverse effects, and routine supplementation without evidence of deficiency could obscure the underlying cause of inadequate treatment response [24,154,158]. Controlled clinical trials are necessary to ascertain whether copper co-supplementation benefits specific high-risk groups, including patients receiving prolonged high-dose iron therapy, individuals with marginal copper intake, and patients with persistent anemia despite adequate iron storage.

Until more robust human evidence is available, individualized iron therapy, accompanied by dose justification, treatment-duration control, follow-up evaluation, and selective copper-related assessment when clinically indicated, represents the most defensible clinical approach. Copper supplementation should be considered only after evaluating dietary intake, biochemical findings, clinical risk factors, and alternative explanations for the inadequate hematological response.

## 9. Knowledge Gaps and Future Directions

### 9.1. Priorities for Human Intervention Studies

Human intervention studies must be rigorously designed to assess whether iron supplementation induces or exacerbates copper deficiency. Essential design elements should include baseline stratification of copper levels, precise quantification of dietary copper and zinc intake, clinically relevant formulations and dosages of iron, adequate treatment duration, and repeated assessments during both the supplementation and recovery phases [159,160,161,162]. Studies should prespecify marginal copper status as a potential effect modifier, rather than relying solely on group mean serum copper levels.

### 9.2. Development and Validation of Functional Copper Biomarkers

The development of copper biomarkers is a priority. Candidate panels should evaluate serum copper, ceruloplasmin protein, ceruloplasmin ferroxidase activity, erythrocyte CCS, SOD1 protein and activity, hematologic indices, and inflammatory markers against controlled copper depletion and repletion [149,160,162,163,164,165,166]. Experimental studies by Prohaska and colleagues demonstrated that erythrocyte CCS abundance and the CCS/SOD1 ratio respond to copper depletion and repletion in rodent models, supporting their further evaluation as candidate biomarkers; however, their analytical performance, within-person variability, reference intervals, and diagnostic thresholds remain to be established in humans [163,164,165,166].

### 9.3. Methodological Priorities for Animal Studies

Animal studies should distinctly separate variables such as iron formulation, dosage, copper content, growth stage, and duration. Carbonyl iron models reporting growth impairment, anemia, organ enlargement, or metabolic injury should include measurements of tissue copper, serum copper, ceruloplasmin protein and ferroxidase activity, hephaestin, and copper rescue groups [122,134,167,168]. These measurements are essential to determine whether a phenotype results from direct iron toxicity, secondary copper limitation, or a combination of both.

## 10. Conclusions

Iron supplementation remains essential for the prevention and treatment of iron deficiency, but current human evidence does not demonstrate a uniform effect on copper status. Clinical studies have reported decreases, no significant changes, and occasional increases in circulating copper-related indices, indicating that responses may vary according to baseline copper status, iron dose, treatment duration, physiological demand, inflammation, and biomarker selection. Available evidence is therefore insufficient to conclude that routine iron supplementation consistently induces copper deficiency. Nevertheless, prolonged or high-dose iron exposure may warrant closer consideration in individuals with marginal copper status or other risk factors for copper insufficiency. Appropriate iron therapy should remain the priority, while unnecessary high-dose exposure should be avoided and copper status considered when hematologic responses are atypical or copper insufficiency is suspected.

Mechanistic evidence from experimental animal models is more consistent and indicates that high dietary iron exposure can impair copper homeostasis. Excessive iron intake has been associated with reduced copper availability, impaired copper-dependent ferroxidase activity, restricted iron mobilization, and tissue iron sequestration despite abundant iron stores. These disturbances may contribute to anemia, impaired growth, cardiac enlargement, and metabolic abnormalities. However, findings from studies using high-dose carbonyl iron without direct assessment of copper status should be interpreted as supportive rather than definitive evidence of copper-mediated effects. Future studies should define the iron doses and exposure durations that compromise copper homeostasis, determine the translational relevance of these mechanisms in humans, and validate sensitive biomarker panels for detecting marginal or functional copper deficiency.

## Figures and Tables

**Figure 1:**
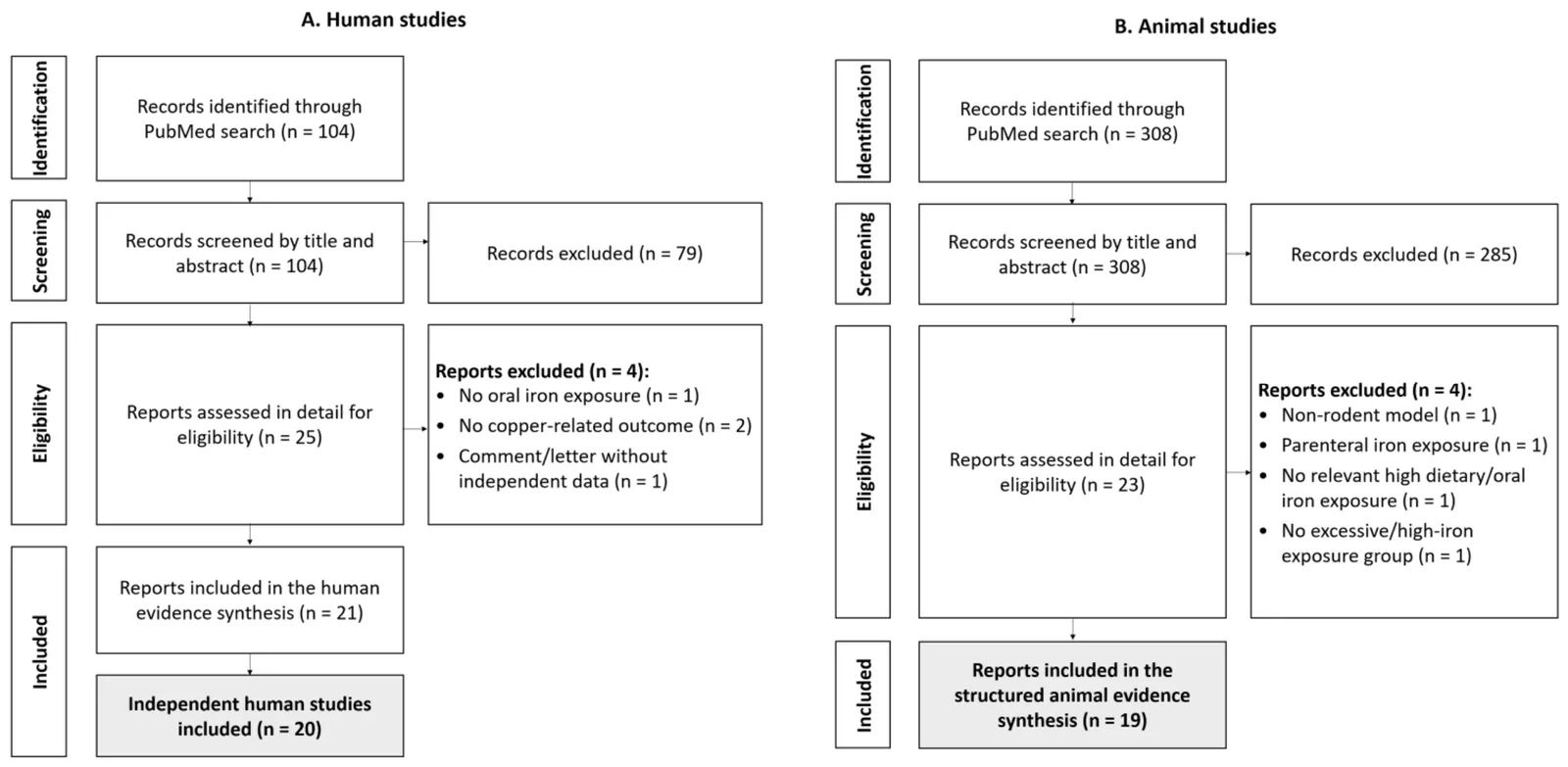
PRISMA-style flow diagram of study identification and selection. The diagram summarizes the identification, screening, eligibility assessment, and inclusion of human and animal studies examining oral or dietary iron exposure in relation to copper homeostasis. The human search yielded 104 records, of which 21 reports representing 20 independent studies were included in the evidence synthesis. The animal search yielded 308 records, of which 19 reports were included in the structured animal evidence synthesis. Additional studies identified through reference-list screening were used as contextual evidence where relevant but were not included in the primary structured search counts.

**Figure 2:**
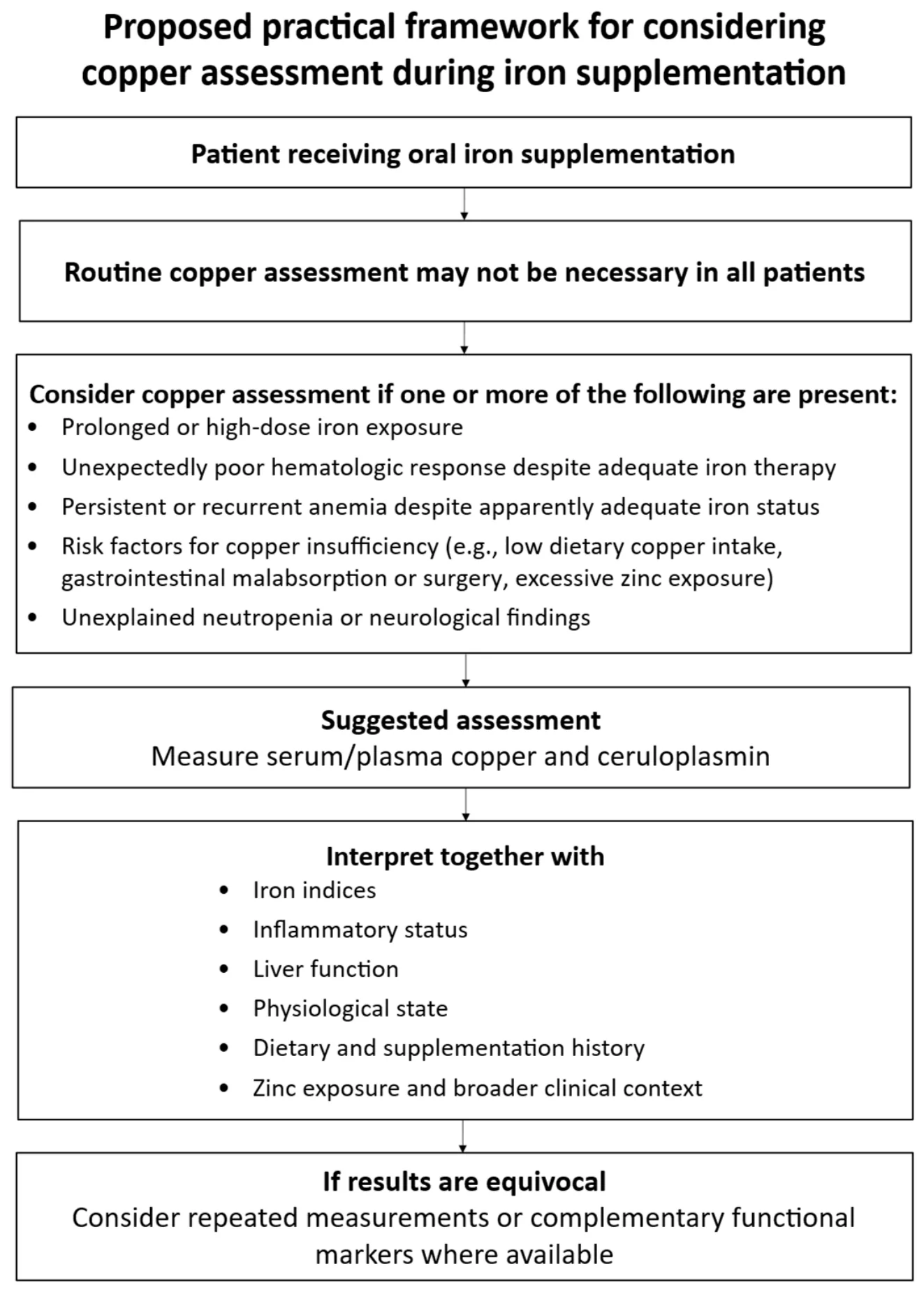
Proposed practical framework for considering copper assessment during oral iron supplementation. Copper assessment may be considered in patients with prolonged or high-dose iron exposure, an unexpectedly poor hematologic response, persistent anemia despite adequate iron status, recognized risk factors for copper insufficiency, or unexplained neutropenia or neurological findings. Serum or plasma copper and ceruloplasmin should be interpreted together with relevant clinical and biochemical factors. This framework is intended as a practical aid rather than a formal clinical guideline, and specific thresholds for high-dose or prolonged iron exposure have not been established.

## Data Availability

No new data were created or analyzed in this study. Data sharing is not applicable to this article.

## Abbreviations

The following abbreviations are used in this manuscript:

ATP7A	ATPase copper transporting alpha
CCS	copper chaperone for superoxide dismutase 1
CDA	copper-deficiency anemia
Cp	ceruloplasmin
CTR1	copper transporter 1
DMT1	divalent metal transporter 1
Epo	erythropoietin
Erfe	erythroferrone
FeSO_4_	ferrous sulfate
IDA	iron-deficiency anemia
LDL	low-density lipoprotein
